# New Appliances and Things Medical

**Published:** 1898-12-10

**Authors:** 


### NEW APPLIANCES AND THINGS MEDICAL.

[We shall be glad to receive, at our Office, 28 & 29, Southampton Street, Strand, London, W.C., from th manufacturers, specimens of all new preparations and appliances which may be brought out from time to time.]

### EUGOL TOOTH POWDER.

(Bayard, Sons, and Bayard, 26, Bridle Lane, Golden Square, W.)

Eugol tooth powder contains the antiseptic eugol combined with a suitable detergent and anti-acid substance, and is a very pleasant dentifrice. Examination shows that it contains no ingredient that is likely to cause any deleterious action on the enamel of the teeth, but rather such as would ensure a corrective to the acidity of the mouth, while its aromatic constituents would serve as a pleasant and efficacious disinfectant and render the breath sweet and fragrant. The powder is contained in a glass bottle fitted with a cork supplied with a sliding cap or sprinkler, so that the powder can be shaken on the tooth brush as required.

### CREOSOTAL. DUOTAL.

(Farbenfabriken, Vorm. F. Bayer and Co., Elberfeld, 19, St. Dunstan's Hill, E.C.)

The preparation of definite compounds of creasote and guaiacol is a great advance over the ordinary forms of these important remedial agents. Creosotal is a liquid substance, consisting of the phenol carbonates of creasote. It is of honey consistency, almost without odour, has a slightly bitter taste, and is non-caustic. It is insoluble in water, but it can be administered by admixture with yolk of egg or with alcoholic liquids. Creosotal has been used with success in cases of tuberculosis by increasing the appetite, strength, and weight. Duotal, a carbonate of guaiacol, is a white crystalline powder, insoluble in water and without taste or smell. It does not irritate the mucous membrane like creasote or guaiacol. Duotal has given good results in the treatment of tuberculosis and other affections of the respiratory organs. The daily dose is 8 grains, increasing to 6 grains.

### HEROIN.

(Farbenfabriken, Vorm. F. Bayer and Co., Elberfeld, 19, St. Dunstan's Hill, E.C.)

Heroin is a suggested substitute for morphine and its preparations and other narcotics. It is a chemical derivative of morphine. It is stated that patients with weak hearts do not experience disagreeable symptoms with heroin as with morphia. Moreover, the dose is smaller and it does not cause constipation, whilst it is as efficacious as codeine and less toxic in its action. Heroin being insoluble in water is best administered either in the form of powder mixed with sugar or dissolved in alcoholic liquids or in water acidulated with acetic acid. In doses of five milligrams three times a day it is said to have given excellent results in cases of bronchitis, pharyngitis, laryngitis, and in catarrh of the lungs in phthisical patients.

### VEGETALINE.

(Patent Vegetaline Company, 25, St. Bees Street, Moss Side, Manchester.)

This is a most ambitious preparation. By its use, its supporters contend, milk can be kept perfectly sound for three days, cream for a week, and butter for a month, in any temperature without loss of flavour. "The basis of vegetaline being the food of the cow, the fluid so readily assimilates with milk that it becomes untraceable by analysts." Whether it is altogether desirable that milk and its products should be able "to keep" this length of time is a serious question, unless there is a practical certainty that no pathogenic bacteria can survive the exposure to "Vegetaline." Milk that is not sour may be far more injurious to health than the most rancid specimen. Our knowledge of vegetaline must be greatly extended before we can express a favourable opinion.

